# Azygous Vein Coil Implantation in Left Ventricular Assist Device Patients: A Hands-on Approach

**DOI:** 10.19102/icrm.2021.121002

**Published:** 2021-10-15

**Authors:** Vijaywant Brar, Susan O’Donoghue, Seth J. Worley

**Affiliations:** ^1^MedStar Heart and Vascular Institute, Washington, DC, USA

**Keywords:** Azygous vein, implantable cardioverter-defibrillator, left ventricular assist device, ventricular tachycardia

## Abstract

Recently, there have been reports of left ventricular assist device (LVAD) patients presenting with multiple ineffective implantable cardioverter-defibrillator (ICD) shocks. In such patients, the placement of an azygous vein coil by providing an alternative anteroposterior trajectory of the electrical shock vector can enable successful defibrillation. This review discusses a hands-on approach to azygous vein coil implantation. Additionally, we compare our tools and technique to those that have been previously described by other operators. From 2018 to 2021, eight patients were identified who underwent azygous vein coil implantation at MedStar Washington Hospital Center using a specific technique and tools. Demographic and procedural data were obtained by a retrospective review of patient charts, procedure logs, fluoroscopy, and venography performed during coil implantation. The indication for azygous vein coil implantation was ineffective ICD shocks in seven patients. The presenting rhythm was ventricular fibrillation in six (75%) cases and sustained ventricular tachycardia in two (25%) cases. Using the approach described, we were able to successfully implant an azygous vein coil in all eight (100%) patients. There were no procedure-related complications. Postimplantation, defibrillation threshold (DFT) testing was successfully performed in six of eight (75%) patients. One patient failed DFT testing despite placement of an azygous vein coil. In another patient, DFT testing was not performed because the patient was in atrial fibrillation and was not systemically anticoagulated. In conclusion, the placement of an azygous vein coil in LVAD patients with failed ICD shocks using the tools and technique described in this report is safe and highly efficacious (successful in 100% of cases).

## Introduction

An elevated defibrillation threshold (DFT) or ineffective shocks are rarely encountered in the contemporary era of primary prevention implantable cardioverter-defibrillator (ICD) implantation, owing to the effectiveness of modern devices.^1^ However, reports of left ventricular assist device (LVAD) patients presenting with multiple ineffective ICD shocks have emerged recently.^2^ Methods to lower the DFT in such patients include optimizing their hemodynamics, correcting any underlying electrolyte abnormalities, eliminating any membrane-active drugs (eg, amiodarone) that could potentially raise the DFT, and implanting additional ICD leads to provide an alternative electrical shock vector. Potential targets for the latter include the superior vena cava (SVC), the subclavian vein, the coronary sinus (CS), and the azygous vein.

Implanting a defibrillation coil in the azygous vein to lower the DFT was first described by Cesario et al. in 2004.^3^ Recently, this strategy was shown to facilitate effective defibrillation in LVAD patients with previously failed ICD shocks.^2^ One can only expect that the need for such interventions will continue to rise with the growing prevalence of patients with LVADs.^4^ The purpose of this report was to describe a hands-on approach to azygous vein coil implantation using our safe and highly efficacious technique. Additionally, we highlight the advantages of using our technique in comparison with that described by other operators in the past.

## Methods

From 2018 to 2021, eight patients were identified in whom azygous vein coil implantation was attempted at MedStar Washington Hospital Center using a specific technique and tools. As per institutional guidelines, all patients provided written informed consent for the implant procedure and subsequent DFT testing. The study was approved by the MedStar Health Research Institute Institutional Review Board, which consented to the use of anonymized medical information for this report. Patients’ electronic medical records, including clinical notes, procedure notes, fluoroscopy, and venography performed during coil implantation, were reviewed.

### Azygous vein coil implantation technique: a hands-on approach

Prior to the actual azygous vein coil–implantation procedure, process optimization^5^ was performed, including preprocedural hydration and elevation of the patient’s legs to increase the central venous pressure **(Video 1)**. The axillary vein was accessed with a 21-gauge, echo-enhanced micropuncture needle by sticking while the contrast was flowing through the target vein (10–20 mL of full-strength contrast, followed by 30–50 mL of flush with normal saline). A stiffened micropuncture dilator and a 5-French (Fr) catheter (Merit S-MAK; Merit Medical, South Jordan, UT, USA) were advanced over the 0.018-in wire. The stiffened dilator was removed, and a 0.035-in glidewire was introduced through the 5-Fr catheter into the subclavian vein. A height-adjustable table was placed perpendicular to the patient; this table orientation allows for long wires, catheters, and sheaths to remain in their natural orientation as they exit the body, thereby minimizing the risk of them falling off the table and eliminating unnecessary bends and curves. A standard vein selector (a braided catheter with a 5-Fr outer diameter, 75-cm-long with a soft tapered tip: Merit Medical) was advanced over the 0.035-in glidewire to the SVC/right atrial junction. The glidewire was removed, and a contrast injection system consisting of a 30-mL contrast reservoir syringe, a 10- to 12-mL control syringe, a three-way stopcock, a 12- to 18-in tube with male and female ends, and a Y-adapter with a hemostatic valve and a rotating hub was assembled and connected to the standard vein selector. Then, the fluoroscopy camera was positioned in the left anterior oblique (LAO) 30° angulation.

**Figure 1:**
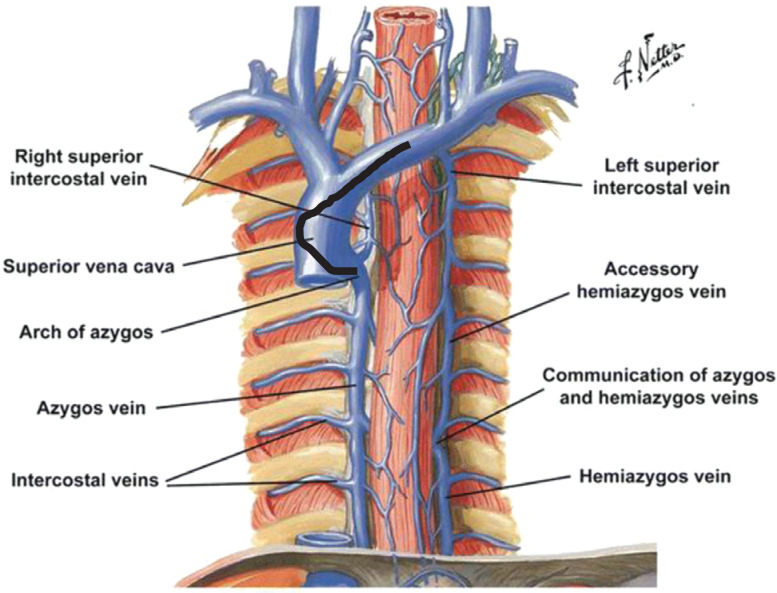
Anatomy of the azygous vein. The black line indicates a JL-3.5 catheter as it engages the azygous vein.

**Figure 2:**
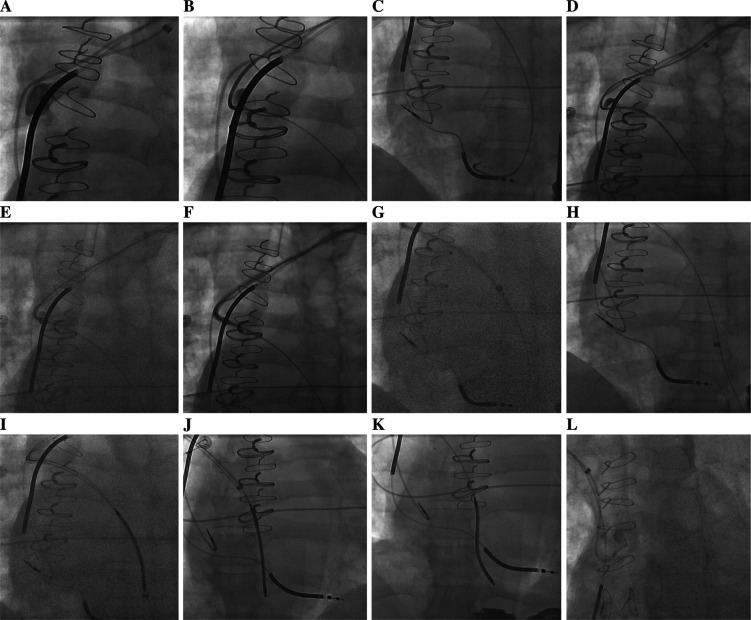
Azygous vein coil implantation technique. **A–K:** Left-sided technique. **L:** Right-sided technique.

Generally, the azygous vein starts at the level of the first and second lumbar vertebrae and arises from the union of lumbar veins and the right subcostal vein. It courses along the right vertebral column and arches posteriorly over the right main bronchus to empty into the SVC **(Figure 1)**. Hence, we began searching for the azygous vein at the beginning of the SVC using puffs of contrast rather than the “poke and pray” wire technique. The azygous vein was located using gentle contrast injections through the standard vein selector. When possible, cannulating the azygous vein with a vein selector was preferred, as this device is much softer and easier to advance into the azygous vein than the Judkins left 3.5 (JL-3.5) diagnostic catheter **(Figure 2A)**. If we had difficulty locating the azygous vein using the standard vein selector, we switched to the JL-3.5 diagnostic catheter attached to the contrast-injection system. As the azygous vein is a posterior structure, we applied a counterclockwise torque to the JL-3.5 diagnostic catheter to locate it. After engaging the azygous vein, a 0.035-in glidewire was advanced as far as possible **(Figure 2B)**. The standard vein selector or the JL-3.5 diagnostic catheter was advanced deep into the azygous vein over the glidewire **(Figures 2C and 2D)**. The glidewire was then exchanged with a 180-cm, 0.035-in Amplatz extra-stiff wire (Cook Medical, Bloomington, IN, USA). Ideally, the Amplatz extra-stiff wire was deposited below the level of the diaphragm. The Worley sheath (9-Fr inner diameter peel-away platform; Merit Medical) along with its hand-shaped/curved stylet was advanced over the Amplatz wire, until the tip of the stylet was at the origin of the azygous vein. The precurved Worley sheath reduced kinking as the sheath negotiated the curves at the brachiocephalic vein–SVC–azygous vein intersection **(Figure 2F)**. The Worley sheath was advanced over the Amplatz extra-stiff wire, deep into the azygous vein **(Figure 2H)**. The coil with the stylet in place was advanced through the Worley sheath adjacent to the Amplatz wire and placed deep in the azygous vein posterior to the heart **(Figure 2I)**. The Amplatz wire was then removed and the Worley sheath was subsequently peeled away, maintaining a stable position of the coil **(Figure 2J)**. The stylet of the azygous coil was then removed **(Figure 2K)** and DFT testing was performed.

## Results

The mean age of our study participants was 51 years, and the majority of them were men (n = 6; 75%). All study participants suffered from severe left ventricular (LV) systolic dysfunction and had an LVAD in place. Additionally, seven patients had pre-existing ICDs, which were placed before their LVAD implantation surgery. Six (75%) patients had a history of successful ICD shocks prior to LVAD implantation surgery. The indication for azygous vein coil implantation in all cases was failed defibrillation. The presenting rhythm was ventricular fibrillation (VF) in six (75%) cases and sustained ventricular tachycardia (VT) in two (25%) cases. Supported by their LVADs, all patients were awake at the time of VT/VF, and the majority of them (n = 5; 62%) experienced more than four consecutive ineffective ICD shocks prior to their presentation **(Table 1)**.

In all eight patients, an azygous vein coil was successfully implanted using process optimization plus the tools and technique described **(Table 2)**. No complications related to the azygous vein coil implantation procedure occurred. Additionally, no changes in the parameters of other nontargeted leads, including sensing, capture threshold, and impedance leads, were noted.

DFT testing was performed in seven patients. One patient was noted to be in atrial fibrillation and was not receiving therapeutic anticoagulation at the time of the implant procedure, so DFT testing was not performed. In one patient, DFT testing was not successful, despite the azygous vein coil implantation and using the highest energy generator available at the time (45 J; Biotronik, Berlin, Germany). In this patient, we additionally placed a subcutaneous coil from the left subclavian vein to the right ventricle (RV). VF was again induced, and the patient was unsuccessfully shocked from the azygous vein coil to the intravascularly placed subcutaneous coil. Due to the lack of other options, this patient was referred for an urgent cardiac transplantation workup.

## Discussion

Most patients with severe stage D systolic heart failure necessitating LVAD therapy have ICDs implanted before their LVAD surgery. Although ICD therapy has not been conclusively shown to provide mortality benefit in LVAD patients,^6,7^ owing to the high incidence of ventricular arrhythmias,^8,9^ therapies including shocks are programmed in most patients. Recently, an increasing number of LVAD patients have been reported to present with multiple ineffective ICD shocks.^2^ The high DFTs in LVAD patients may be secondary to the severity of LV dysfunction or the post-LVAD rise in DFT.^10,11^ The post-LVAD rise in DFT may be due to changes in the cardiac geometry and shunting of the electrical shock due to vector shifts caused by the introduction of an intrathoracic metal.^10^ Unfortunately, most patients with LVADs are fully conscious while being shocked by their ICD repeatedly as their hemodynamics are supported by the LVAD, which can lead to major psychological trauma.

Frequently used but often ineffective options for LVAD patients with appropriate but failed ICD shocks include: (1) programming changes including altering the vector polarity and adjusting the tilt and pulse width of the biphasic shock^12^; (2) repositioning of the RV ICD lead; (3) upgrading to a dual-coil ICD system; (4) use of a stronger energy generator; (5) the addition of a second defibrillator coil in a different location (eg, subclavian vein or CS); and (6) implantation of a subcutaneous ICD. Although there have been case reports of implantation of subcutaneous ICDs in patients with an LVAD, these devices are not considered optimal for such patients.^13,14^ Some potential issues include the proximity of the ICD pocket site to the LVAD, electromagnetic interference from the LVAD, and the inability to deliver antitachycardia pacing. By comparison, placement of an azygous vein coil, by providing an alternative anteroposterior trajectory of the electrical shock vector, can be very effective in lowering the DFT.

In our series, the RV ICD lead position and parameters, including shock impedance, were within the normal limits for all patients; hence, repositioning the RV lead would likely be of minimal benefit. Four patients previously had a well-positioned SVC coil (dual-coil RV lead). Additionally, three patients previously had biventricular ICDs. Even though LV pacing is often turned off in patients with LVADs, the presence of a pacing lead in the CS makes the addition of an ICD lead in the CS more challenging. Therefore, our approach was to implant an azygous vein coil to provide an anteroposterior shock vector. We were able to successfully place the azygous vein coil in all eight (100%) of our patients. Additionally, we placed a high-energy generator in four (50%) patients.

The largest case series of azygous vein coil implantations (n = 10 patients) to date was published by Cooper and Smith^15^ in 2009. In their series, these authors noted a success rate of more than 90% in azygous vein coil implantation using their tools and technique. Their failure to implant the coil was secondary to their inability to advance the long sheath around the curve into the azygous vein. In another series, Seow et al.^16^ reported on three patients and claimed a success rate of 66%. In comparison, we were able to implant the azygous vein in all eight (100%) LVAD patients in whom it was attempted using our tools and technique. Additionally, our technique has some cardinal differences from those previously described **(Table 3)** as follows.

**Figure 3:**
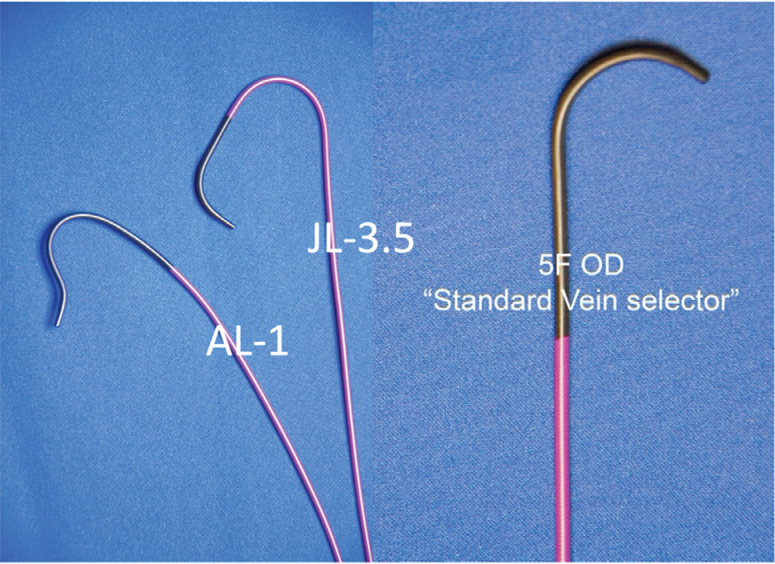
Catheters used for engaging the azygous vein: AL-1, JL-3.5, and standard vein selector. AL-1: Amplatz left 1 diagnostic catheter; JL-3.5: Judkins left 3.5 diagnostic catheter; OD: outer diameter.

First, (1) we emphasized the importance of preprocedural process optimization, including prehydration and height-adjustable perpendicular table position.^5^ (2) When implanting from the left side, a JL-3.5 catheter is better suited to engage the azygous vein when compared to the JR-4 catheter as described by others.^15,16^ The secondary and tertiary curves direct the JL-3.5 catheter inferiorly, and the primary curve engages the azygous vein with a counterclockwise torque **(Figure 3)**. Also, (3) we recommend using the Amplatz left 1 (AL-1) catheter to engage the azygous vein when implanting from the right side **(Video 2)**. Cooper and Smith^15^ described using the right mainstem bronchus in the anteroposterior fluoroscopy view as a reference starting point for locating the azygous vein. In contrast, (4) we encourage positioning the catheter in the SVC, central to the origin of the left brachiocephalic vein, and then using a gentle pullback and counterclockwise torque to point the catheter tip toward the left side of the patient while imaging in the left anterior oblique 30° view. Further, (5) we strongly encourage using gentle contrast injections through specifically shaped catheters to visualize the origin of the azygous vein rather than probing with a wire to locate the azygous vein. Performing “poke and pray” with a wire, as compared with contrast-guided catheter engagement, adds time to the procedure and makes it more challenging to locate the azygous vein. (6) Once the azygous vein is engaged and a catheter (standard vein selector, eg, JL-3.5 or AL-1) is advanced to the level of the diaphragm over the glidewire, we recommend routinely exchanging the glidewire with an Amplatz wire, then advancing the long, precurved sheath with a hand-curved dilator over the Amplatz wire to prevent kinking of the sheath at the SVC–azygous vein junction. Finally, (7) in contrast to previous reports, we recommend advancing the ICD coil adjacent to the retained Amplatz wire, which should keep the long sheath from kinking.

## Conclusion

The prevalence of LVAD patients is expected to continue to rise in the coming decades. Azygous vein coil implantation is probably the most effective bailout strategy for such patients who present with ineffective ICD shocks. Azygous vein coil placement can be accomplished safely with a high success rate when a standardized meticulous implantation technique such as the one described in this review is followed.

## Figures and Tables

**Video 1:** Azygous vein coil implantation technique from the left side.

**Video 2:** Azygous vein coil implantation technique from the right side.

**Table 1:** Baseline Patient Characteristics and Clinical Presentation

Age (years), mean ± standard deviation	51 ± 11
Female sex, n (%)	2 (25)
Hypertension, n (%)	8 (100)
Diabetes, n (%)	3 (38)
Dyslipidemia, n (%)	4 (50)
Smoker, n (%)	4 (57)
Etiology of cardiomyopathy, n (%)	
Ischemic cardiomyopathy	1 (12)
Nonischemic cardiomyopathy	7 (88)
LVAD type, n (%)	
HeartMate III	4 (50)
HeartMate II	1 (12)
HeartWare	3 (38)
Type of ICD, n (%)	
None	1 (12)
Single coil	3 (38)
Dual coil	4 (50)
Indication for ICD implantation, n (%)	
Primary prevention	7 (88)
Secondary prevention	1 (12)
History of successful ICD shocks pre-LVAD	6 (75)
Presenting arrhythmia, n (%)	
Ventricular fibrillation	6 (75)
Sustained ventricular tachycardia	2 (25)
Number of ineffective ICD shocks at presentation, n (%)	
0–3	3 (38)
3–6	5 (62)

ICD: implantable cardioverter-defibrillator; LVAD: left ventricular assist device.

**Table 2:** Procedural Interventions and Outcomes

Patient No.	Subclavian Fibroplasty Performed	Side of Implant	Catheter Used	Lead Implanted	Success of DFT Testing	Energy at Which DFT Testing Was Performed
1	Yes	Right	AL-1	Medtronic* SQ coil 6996SQ-58	Successful	30 J
2	Yes	Left	Std vein selector	Medtronic 6937A-58	Successful	45 J
3	No	Left	Std vein selector	Medtronic 6937A-58	Failed	45 J
4	No	Left	JL-3.5	Medtronic 6937A-58	Not performed	
5	No	Left	JL-3.5	Medtronic 6937A-58	Successful	30 J
6	No	Left	JL-3.5	Medtronic 6937A-58	Successful	30 J
7	No	Left	JL-3.5	Medtronic 6937A-58	Successful	45 J
8	Yes	Left	JL-3.5	Medtronic 6937A-58	Successful	45 J

AL-1: Amplatz left 1 diagnostic catheter; DFT: defibrillation threshold; JL-3.5: Judkins left 3.5 diagnostic catheter; Std: standard; VF: ventricular fibrillation; VT: ventricular tachycardia.

*Medtronic (Minneapolis, MN, USA)

**Table 3:** Differences in the Equipment and Azygous Vein Coil Implantation Technique

	Cooper and Smith^15^	Brar et al.
Tools	• A 9-Fr peel-away long sheath • A 6-Fr JR-4 used to engage the azygous vein • A Wholey wire advanced into the azygous vein	• A 9-Fr Worley standard sheath • A 6-Fr JL-3.5 and a 6-Fr standard vein selector used to engage the azygous vein from the left • A 6-Fr AL-1 used to engage the azygous vein from the right • A 0.035-in glidewire advanced into the azygous vein • The glidewire exchanged with a 0.035-in Amplatz wire
Technique	• Fluoroscopy camera positioned in AP projection • Right mainstem bronchus used as a reference starting point for locating the azygous vein • Probing with the wire to locate the azygous vein • Long sheath advanced into the azygous vein over the wire without the dilator	• Emphasized preprocedural process optimization • Fluoroscopy camera positioned in steep LAO 30° • Catheter advanced to the SVC–RA junction and azygous vein engaged with a gentle pullback and counterclockwise torque • Use of gentle contrast injections to locate the azygous vein • Once the catheter is deep in the azygous vein, the glidewire is routinely exchanged with an Amplatz wire • ICD lead is inserted adjacent to the Amplatz wire

AL-1: Amplatz left 1 diagnostic catheter; AP: anteroposterior; ICD: implantable cardioverter-defibrillator; JL-3.5: Judkins left 3.5 diagnostic catheter; JR-4: Judkins right 4 diagnostic catheter; LAO: left anterior oblique; SVC–RA: superior vena cava–right atrium.
